# Cardiac microstructural alterations in immune-inflammatory myocardial disease: a retrospective case-control study

**DOI:** 10.1186/s12947-022-00279-0

**Published:** 2022-04-04

**Authors:** Alan C. Kwan, Gerran Salto, Trevor-Trung Nguyen, Elizabeth H. Kim, Eric Luong, Pranoti Hiremath, David Ouyang, Joseph E. Ebinger, Debiao Li, Daniel S. Berman, Michelle M. Kittleson, Jon A. Kobashigawa, Jignesh K. Patel, Susan Cheng

**Affiliations:** 1 Department of Cardiology, Smidt Heart Institute, Cedars-Sinai Medical Center, Los Angeles, CA USA; 2 Division of Cardiovascular Medicine, Brigham and Women’s Hospital, Boston, MA USA; 3 Framingham Heart Study, Framingham, MA USA; 4 Division of Cardiology, Johns Hopkins Hospital, Baltimore, MD USA; 5 Biomedical Imaging Research Institute, Cedars-Sinai Medical Center, Los Angeles, CA USA; 6 Department of Imaging, Cedars-Sinai Medical Center, Los Angeles, CA USA

**Keywords:** Cardiac microstructure, Myocarditis, Transplant rejection, Echocardiography

## Abstract

**Background:**

Immune-inflammatory myocardial disease contributes to multiple chronic cardiac processes, but access to non-invasive screening is limited. We have previously developed a method of echocardiographic texture analysis, called the high-spectrum signal intensity coefficient (HS-SIC) which assesses myocardial microstructure and previously associated with myocardial fibrosis. We aimed to determine whether this echocardiographic texture analysis of cardiac microstructure can identify inflammatory cardiac disease in the clinical setting.

**Methods:**

We conducted a retrospective case-control study of 318 patients with distinct clinical myocardial pathologies and 20 healthy controls. Populations included myocarditis, atypical chest pain/palpitations, STEMI, severe aortic stenosis, acute COVID infection, amyloidosis, and cardiac transplantation with acute rejection, without current rejection but with prior rejection, and with no history of rejection. We assessed the HS-SIC’s ability to differentiate between a broader diversity of clinical groups and healthy controls. We used Kruskal-Wallis tests to compare HS-SIC values measured in each of the clinical populations with those in the healthy control group and compared HS-SIC values between the subgroups of cardiac transplantation rejection status.

**Results:**

For the total sample of *N* = 338, the mean age was 49.6 ± 20.9 years and 50% were women. The mean ± standard error of the mean of HS-SIC were: 0.668 ± 0.074 for controls, 0.552 ± 0.049 for atypical chest pain/palpitations, 0.425 ± 0.058 for myocarditis, 0.881 ± 0.129 for STEMI, 1.116 ± 0.196 for severe aortic stenosis, 0.904 ± 0.116 for acute COVID, and 0.698 ± 0.103 for amyloidosis. Among cardiac transplant recipients, HS-SIC values were 0.478 ± 0.999 for active rejection, 0.594 ± 0.091 for prior rejection, and 1.191 ± 0.442 for never rejection. We observed significant differences in HS-SIC between controls and myocarditis (*P* = 0.0014), active rejection (*P* = 0.0076), and atypical chest pain or palpitations (*P* = 0.0014); as well as between transplant patients with active rejection and those without current or prior rejection (*P* = 0.031).

**Conclusions:**

An echocardiographic method can be used to characterize tissue signatures of microstructural changes across a spectrum of cardiac disease including immune-inflammatory conditions.

## Background

Accumulating evidence suggests that immune or inflammatory processes contribute to a wide variety of myocardial disease processes which may transition over time to clinical heart failure [1, 2]. Non-invasive diagnosis of myocardial inflammation through standard methods of positron emission tomography or cardiac magnetic resonance imaging may be limited by cost and accessibility. Acute or chronic inflammatory processes involve tissue alterations at the microstructural level. We have previously developed a method, the high spectrum signal intensity coefficient (HS-SIC), which identifies microstructural changes indicative of fibrosis in multiple clinical scenarios [3–5]. This marker has been primarily applied in populations which have expected fibrosis; therefore, we investigated its application in other populations which represent a larger breadth of myocardial pathology including immune and inflammatory processes. We hypothesized that the HS-SIC may be able to identify other microstructural signatures, such as those found in immune or inflammatory conditions. We evaluated the extent to which the HS-SIC, implemented via routine transthoracic echocardiography, might differentiate a spectrum of microstructural disease and identify inflammatory myocardial processes in the clinical setting.

## Methods

### Study sample

We used retrospective chart query with adjudication to identify patients in our Cedars Sinai Health System with clinical evidence of having 1 of 9 distinct myocardial disease entities at the time of transthoracic echocardiography and compared to a healthy control group. Each disease diagnosis was adjudicated based on ICD9 and ICD10 codes, laboratory data, imaging, and chart review. The following non-overlapping myocardial disease entities were identified with clinical cardiologist adjudication: myocarditis, atypical chest pain or palpitations without medical comorbidities, acute left anterior descending coronary artery STEMI, severe aortic stenosis with normal ejection fraction, acute COVID infection with positive troponin, TTR amyloidosis, and cardiac transplantation status undergoing screening biopsy in 3 groups - active rejection on histopathology, no active rejection but with history of prior rejection, and no active and no history of rejection. All samples except for COVID were drawn from 2019 and prior, avoiding overlap with the COVID population. The atypical chest pain or palpitations excluded any patients with suspected myocarditis or pericarditis. Patients were included in the clinical cohort if they had a contemporaneous transthoracic echocardiogram performed during the index hospitalization for acute conditions (myocarditis, STEMI, COVID); performed in association with a clinical encounter with ICD coding for chronic conditions (severe aortic stenosis, TTR amyloidosis, atypical chest pain, palpitations); or, performed at the time of screening endomyocardial biopsy for cardiac transplantation.

For the healthy control reference group, we selected participants of an established community-based cohort study who had normal range laboratory diagnostics and no known cardiovascular risk factors at the time of transthoracic echocardiography [6]. Identification was performed by screening the sixth and eight cohort visits for patients with normal blood glucose, body mass index, low-density and high-density lipoprotein, triglycerides, creatinine, blood pressure, heart rate, and no history of smoking, cardiopulmonary disease, left ventricular hypertrophy, diabetes, hypertension, or hyperlipidemia medication use for both visits, with the sequential first 20 patients included. All protocols were approved by our institutional review boards; written informed consent was obtained from all participants of the screening cohort, and informed consent was waived for retrospective data analyses for the clinical cohort.

### Myocardial microstructural analysis

**Fig. 1:**
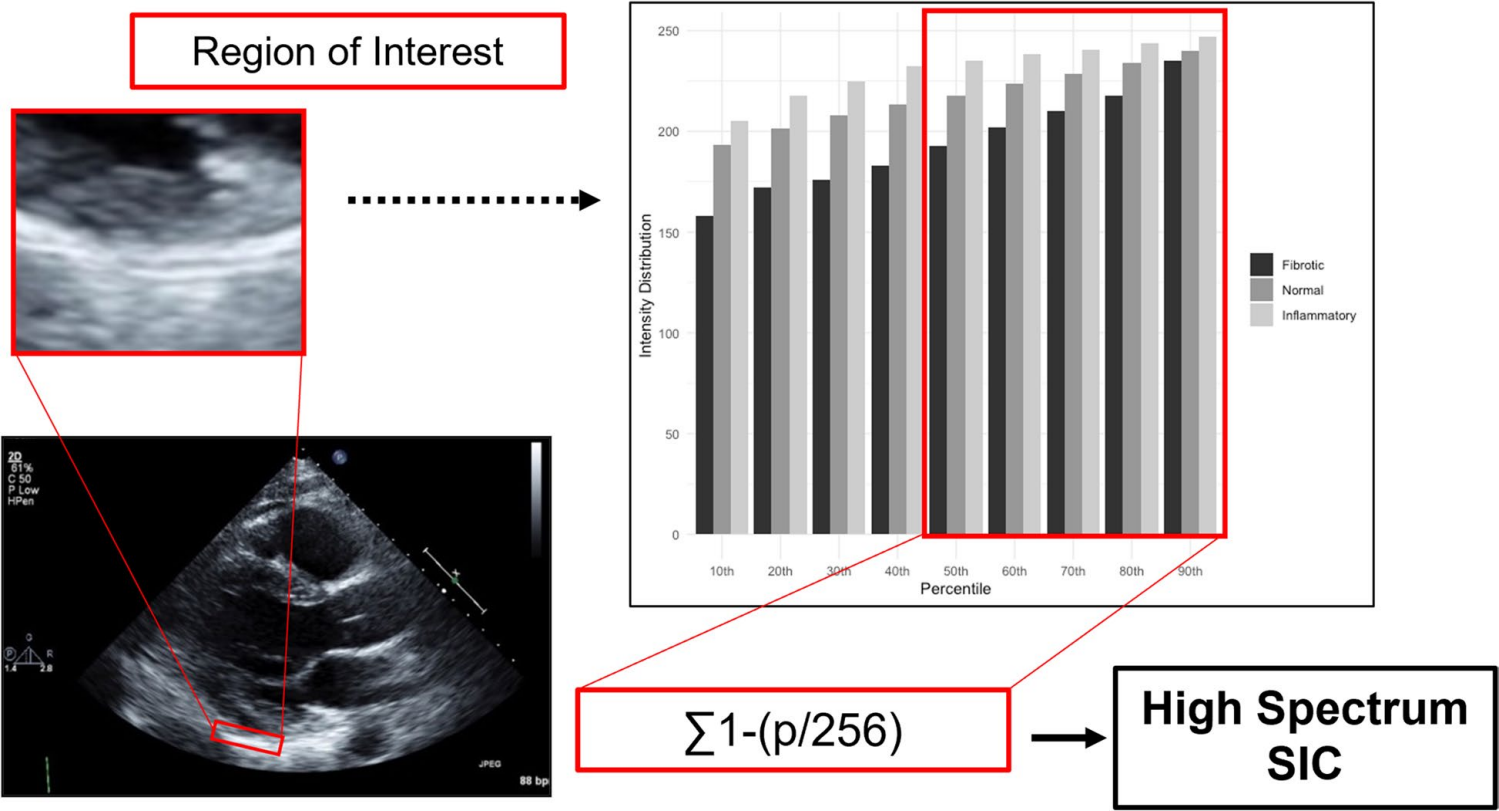
High Spectrum Signal Intensity Coefficient (HS-SIC) analysis method. We analyzed parasternal long axis views acquired with routine protocols. We used ImageJ (v1.53, National Institutes of Health, Bethesda, MD) to select a 5 × 30 pixel region of interest (ROI) at the myocardial-pericardial interface along the inferolateral wall during end-diastole and aligned with at the level of the mitral leaflet tips. We then applied an image analysis macro to quantify the distribution of intensity values within the ROI, ranging from 0 to 256. Values were normalized and integrated across the 50th, 60th, 70th, 80th, and 90th percentiles of signal intensity to generate the HS-SIC

The echocardiographic method for quantifying myocardial microstructure alterations has been detailed previously [3–5]. This texture analysis uses histogram-based radiomic analysis of intensities at the myocardial-pericardial interface and has been validated across different gain settings and for inter- and intra-reader reproducibility in both human and murine models (Fig. 1) [3, 7]. We calculated the high-spectrum signal intensity coefficient (HS-SIC) as the sum of 1-(p/256) where p is the 50th, 60th, 70th, 80th, and 90th percentiles of signal intensity within a region of interest (ROI) placed at the myocardial-pericardial interface in a standard parasternal long axis view. Imaging analysts performing ROI and HS-SIC measurements were blinded to clinical information and diagnoses.

### Statistical analyses

We used Kruskal-Wallis tests to compare HS-SIC values measured in each of the clinical populations with those in the screening control group; we also compared HS-SIC values between the subgroups of cardiac transplantation rejection status. We used R v4.0.0 for all statistical analyses and defined the threshold of significance as a two-tailed *P* < 0.05.

## Results

We identified a total of *n* = 338 patients, with 318 in the clinical cohort and 20 in the screening cohort. For the total study sample, mean age was 49.6 ± 20.9 years and 50% were men, with expected variation in characteristics across clinical diagnosis groups. The number of individuals in each group ranged from 5 in cardiac transplantation patients without either active or prior organ rejection to 127 in the atypical chest pain or palpitations group (Table 1).

**Table 1:** Demographics and High Spectrum Signal Intensity Coefficient (HS-SIC) values for clinical population, mean ± standard error of the mean

Population	N	Age	Male	Diabetes	HTN	HLD	Smoking	CAD	EF	HS-SIC (SEM)	***P***-Value (vs control)
Healthy Controls	20	34.7 ± 7.4	2 (10%)	0 (0%)	0 (0%)	0 (0%)	0 (0%)	0 (0%)	65.7 ± 4.1	0.688 ± 0.074	–
**Patient Cohorts**											
Atypical Chest Pain or Palpitations	127	37.3 ± 12.8	76 (60%)	0 (0%)	0 (0%)	0 (0%)	0 (0%)	0 (0%)	62.5 ± 4.5	0.552 ± 0.049	0.016
Myocarditis	44	40.0 ± 17.1	12 (27%)	7 (15%)	20 (45%)	15 (34%)	11 (25%)	11 (25%)	48.5 ± 16.5	0.425 ± 0.058	0.001
TTR Amyloidosis	25	76.3 ± 9.7	6 (24%)	6 (24%)	17 (68%)	19 (76%)	9 (36%)	6 (24%)	46.6 ± 16.9	0.698 ± 0.103	0.85
STEMI	20	59.1 ± 20.4	6 (30%)	1 (5%)	15 (75%)	16 (80%)	9 (45%)	20 (100%)	40.3 ± 12.9	0.881 ± 0.129	0.30
COVID	39	67.6 ± 16.4	28 (72%)	14 (36%)	25 (64%)	13 (33%)	8 (21%)	7 (18%)	57.2 ± 10.8	0.904 ± 0.116	0.58
Severe Aortic Stenosis	21	82.3 ± 9.7	8 (38%)	5 (25%)	18 (90%)	16 (80%)	8 (40%)	14 (70%)	60.7 ± 11.8	1.116 ± 0.196	0.38
**Transplanted Cohorts**											
No prior or active rejection	5	49.6 ± 14.1	5 (100%)	3 (60%)	3 (60%)	3 (60%)	1 (20%)	1 (20%)	55.8 ± 1.9	1.191 ± 0.442	0.33
Prior but no active rejection	15	54.9 ± 20.6	10 (67%)	8 (53%)	9 (60%)	3 (20%)	4 (27%)	2 (13%)	61.8 ± 3.8	0.594 ± 0.091	0.46
Active rejection	22	48.5 ± 14.4	16 (73%)	10 (45%)	9 (41%)	9 (41%)	4 (18%)	5 (23%)	60.5 ± 6.8	0.478 ± 0.099	0.008
**Total**	338	49.6 ± 20.9	169 (50%)	54 (16%)	116 (34%)	94 (28%)	54 (16%)	66 (20%)	57.4 ± 12.3	0.656 ± 0.032	–

*HTN* Hypertension, *HLD* Hyperlipidemia, *CAD* Coronary artery disease, *EF* Ejection Fraction

**Fig. 2:**
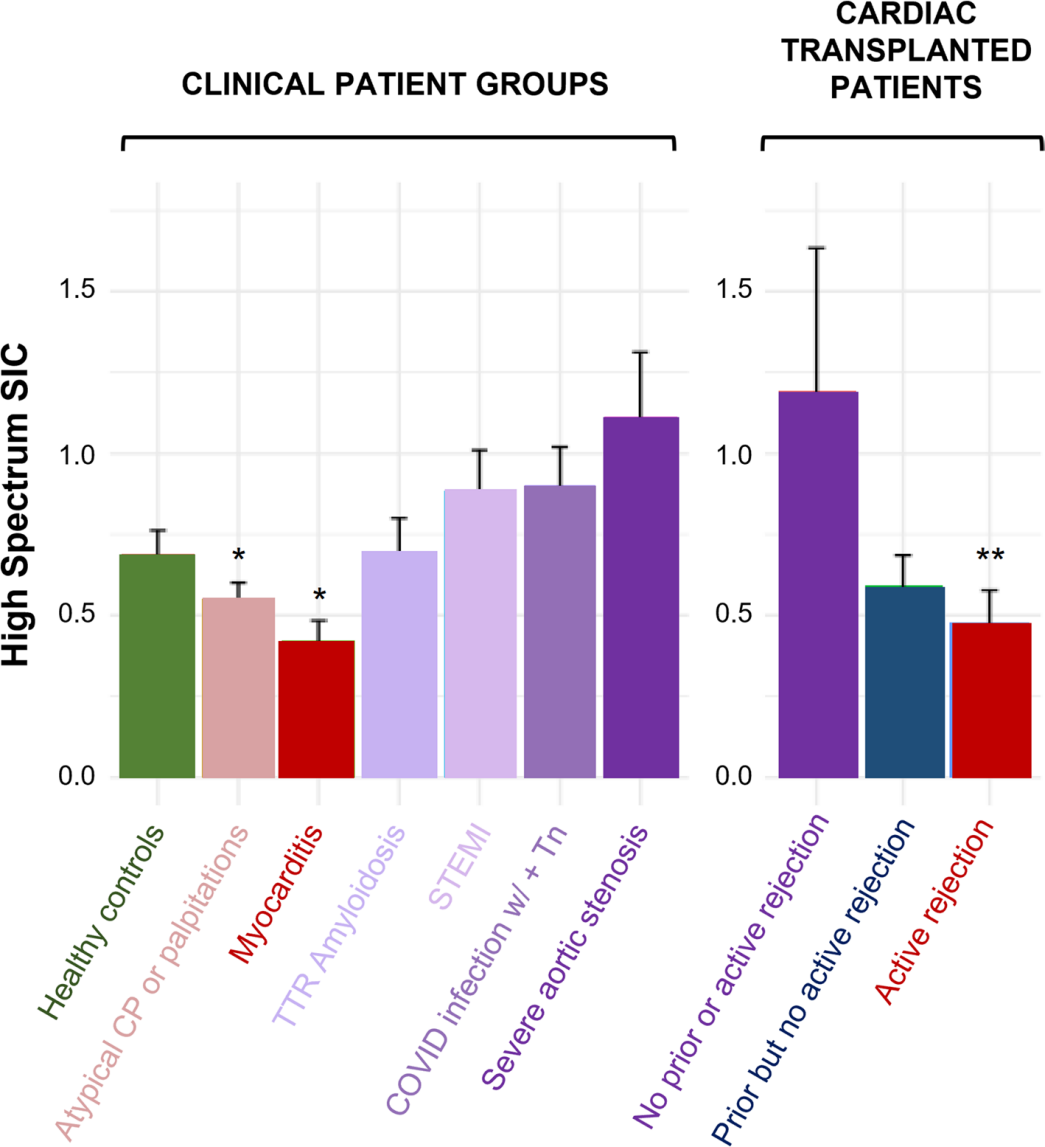
Cardiac microstructural measures across patient groups. High Spectrum Signal Intensity Coefficient (HS-SIC) values for clinical population, with bars shown as mean ± standard error. *P < 0.05 for comparison to Screening Cohort, **P < 0.05 for comparison to Screening Cohort and Transplant without current or historical rejection

We observed that patients with more acute inflammatory conditions (i.e. myocarditis, active atypical cardiac symptoms, active transplant rejection) had lower HS-SIC values, patients with more fibrotic myocardial conditions (i.e. aortic stenosis, transplant without rejection) had higher HS-SIC values (Fig. 2). There were significant differences when comparing HS-SIC values measured in the screening cohort (mean ± SEM: 0.668 ± 0.074, median [IQR]: 0.527 [0.427,0.964]) with the following groups: atypical chest pain or palpitations (mean ± SEM: 0.552 ± 0.049, median [IQR]: 0.316 [0.152,0.727], *p* = 0.016), myocarditis (mean ± SEM: 0.425 ± 0.058, median [IQR]: 0.312 [0.132,0.638], *p* = 0.001), and active rejection (mean ± SEM: 0.478 ± 0.099, median [IQR]: 0.354 [0.209,0.614], *p* = 0.008). We also observed differences within the transplantation subset. Patients with active rejection had lower HS-SIC values versus patients without current or prior rejection (mean ± SEM: 1.191 ± 0.442, median [IQR]: 0.656 [0.578,1.609], *p* = 0.031).

## Discussion

In this clinical proof-of-concept study, we found that a highly accessible echocardiographic texture analysis method was able to distinguish myocardial pathologies from healthy control status. We and others have shown that echocardiographic texture analysis can identify microstructural alterations associated with hypertensive disease [3], preclinical cardiac disease in carriers of hypertrophic cardiomyopathy gene variants [5], and cardiometabolic syndrome [8]. In prior studies, higher HS-SIC values were related to cardiomyocyte hypertrophy or interstitial fibrosis by histopathology or cardiac magnetic resonance [3, 5]. In the current study, we observed higher HS-SIC values in cardiac conditions known to involve similar tissue pathology. Extending from prior work, we observed *lower* HS-SIC values in cardiac conditions known to involve acute immune-inflammatory activity (e.g. myocarditis and acute cardiac transplant rejection). These findings suggest that presence of immune-inflammatory edema or cellular infiltration may lead to changes in acoustic substrate that is detectable and quantifiable. Intriguingly, our findings of mildly reduced HS-SIC levels in patients presenting with atypical cardiac symptoms (e.g. atypical chest pain or palpitations) raises the specter of subclinical inflammation in this setting.

While our results should be interpreted as a proof-of-concept study and requires off-line analysis of echocardiographic images, we believe that this method has potential for future clinical implications. The calculation of the HS-SIC itself is not complex and is software agnostic. If identification of the appropriate view and region of interest can be solved, this measurement could be provided within a clinical interpretation pipeline as supplementary information to clinical readers. This may provide incremental benefit over qualitative estimates of intensity by incorporating both signal intensity and signal distribution information into a quantitative measurement. Preliminary analyses for deep-learning based placement of the ROI are ongoing [9].

Several limitations of our study merit consideration. Because healthy control participants lack indications for clinical echocardiography, our control sample is from a screening dataset with imaging obtained externally. Our study cohort included clinical subgroups that were small in size, precluding multiple statistical testing or multivariable-association analyses. Although the HS-SIC has been validated as measure of myocardial fibrosis [3], we lack a priori histological data for the current study. There were no cases with significant anatomical disruption of the myocardial-pericardial interface (e.g. moderate to large pericardial effusion), and therefore we are uncertain how this may affect the measurement. Given that sampling is only performed in a single region, more patchy inflammatory disease presentation may be less detectable by this method. Further work is needed to validate the HS-SIC as a measure of immune-inflammatory myocardial disease.

## Conclusions

In summary, we found that an accessible echocardiographic imaging method can be used to characterize microstructural changes across a spectrum of myocardial disease including active immune-inflammatory conditions. Given the relatively low cost and wide availability of echocardiography in practice, our results underscore the benefit of potential future immune-inflammatory targeted applications, including early detection of immune-inflammatory cardiac disease (e.g. myocarditis which can be clinically challenging to diagnose), serial monitoring of response to therapies (e.g. in acute cardiac transplant rejection), and longitudinal phenotyping of the progression from immune-inflammatory risk to overt clinical heart failure over time.

## Data Availability

The datasets used during this study are available from the corresponding author on reasonable request.

## Abbreviations

HS-SIC: High spectrum signal intensity coefficient
ROI: Region of interest
